# Criterion validity of the functional status and pain assessment scale versus pain and length of stay in hospitalized adults with SCD

**DOI:** 10.1093/jscdis/yoag012

**Published:** 2026-03-05

**Authors:** Wally R Smith, Daniel M Sop, Yue May Zhang, Margaret S Guy, Rehan Qayyum

**Affiliations:** Division of General Internal Medicine, Department of Internal Medicine, Virginia Commonwealth University, Richmond, VA 23219-0306, United States; Division of General Internal Medicine, Department of Internal Medicine, Virginia Commonwealth University, Richmond, VA 23219-0306, United States; Division of General Internal Medicine, Department of Internal Medicine, Richmond, VA 23219-0306, United States; Division of Hospital Medicine, Department of Internal Medicine, Virginia Commonwealth University, Richmond, VA 23298-0102, United States; Macon & Joan Brock Virginia Health Sciences at Old Dominion University, Norfolk, VA 23501-1980, United States

**Keywords:** SCD, functional pain assessment, criterion validity, length of stay, pain measurement, hospitalization

## Abstract

**Objectives:**

In prior research we demonstrated face, construct, discriminant, and preliminary convergent validity of the newly developed, 10-item Functional Status Pain Assessment (FSPA) scale (0-50) in adult SCD patients hospitalized with VOC. We aimed in this study to further demonstrate criterion validity of the FSPA.

**Methods:**

This was a prospective observational study of 561 daily functional and pain assessments from 91 hospitalized adults with SCD from January 2018 to June of 2019. Principal Component Analysis (PCA) Partial Least Squares (PLS) models determined the relationship between LOS, pain intensity, and FSPA. A Generalized Linear Model (GLM) to quantified the impact of changes in pain intensity on LOS.

**Results:**

The population mean age was 34 ± 12.2 years, and 59.3% were female. A little over half were genotype SS. Neither admission nor discharge FSPA scores were significant determinants of LOS. Specifically, no component or factor of FSPA score on day of admission explained the LOS. In addition, FSPA score on day of discharge explained only 5.69% of the variance in LOS. Similarly, FSPA score explained only 19.66% of the variance in pain intensity on any pain day. There was no relationship between LOS and pain intensity changes from admission to discharge (*P* = .92).

**Conclusions:**

The FSPA score did not demonstrate criterion validity with respect to NRS pain intensity changes from admission to discharge, nor to the LOS for SCD admissions. Instead, unmeasured, perhaps environmental, psychosocial, and economic variables, exerted a far more substantial influence on hospital LOS.

## INTRODUCTION

SCD, the most common set of genetic hemoglobinopathies worldwide,^1^^,^^2^ is caused by single point mutation leading to substitution of valine for glutamic acid in the sixth position of the beta globin chain, *HBB*.^3^ It is associated with frequent, painful vaso-occlusive crises (VOCs) provoking expensive hospitalizations,^4^ and with early morbidity and mortality.^5^

Evaluating when hospitalization for patients with SCD is warranted to manage acute pain, and when discharge from the hospital is appropriate, commonly involves clinicians’ use of a unidimensional Numeric Rating Scale (NRS) of subjective pain intensity. However, pain is a multidimensional experience consisting of intensity, frequency, location, character, and multifaceted pain responses, and is complicated by environmental, psychosocial, and economic drivers.^6^ In addition, unlike children, many adults with SCD experience acute VOC episodes superimposed on years of chronic fluctuating pain, often complicated by long-term opioid exposure.^7^ This chronicity and these complications may alter baseline pain perception and functional capacity, resulting in day-to-day variability independent of hospital course. Therefore, functional tools developed for inpatient pain assessment must account for the unique physiology and behavioral context of chronic and acute-on-chronic pain in SCD.

We developed the Functional Status Pain Assessment (FSPA) scale for daily use in adult SCD patients hospitalized with VOC.^8^^,^^9^ One goal of scale development was to demonstrate feasibility of completing a daily, multi-item, in-hospital survey during a VOC without overburdening patients with questions. A second goal was to show the internal validity of the scale for measuring daily function. A third goal was to determine whether the scale predicted important outcomes like the change in pain score from admission to discharge, or the length of stay.

In prior research we had already demonstrated face, construct, discriminant, and preliminary convergent validity of the 10-item FSPA scale (0-50) in adult SCD patients hospitalized with VOC. We found that the mean score on the 10-item FSPA, scored on a 0-50 scale, was 27 ± 8.0 among all patients for all days when data were submitted. The mean NRS (6.8 ± 1.9) was inversely correlated with the mean FSPA score (*r* = −0.4342, *P* < .0001) among all days and patients.

This gave rise to the present hypotheses prompting these analyses. At minimum, we hypothesized that subjective NRS would decrease from admission to discharge within patients, and that daily FSPA would increase from admission to discharge within patients. If these hypotheses held, we felt patients and clinicians could reasonably use these two variables in their daily negotiations about readiness for hospital discharge. At best, we hypothesized that the FSPA would demonstrate criterion validity against both subjective NRS and length of stay, both giving patients and clinicians a very strong tool to enhance these daily negotiations, and providing a predictive instrument for researchers and clinicians alike.

## MATERIALS AND METHODS

### Sample

We studied 561 assessments from 91 unique patients admitted to one SCD specialized nursing unit from January 2018 to June of 2019 which preferentially selected SCD admissions. Patients who were nonverbal or functionally not able to complete form were excluded. Most patients had more than one hospitalization record. Patients included were those who had a recorded baseline (day of admission) and last day of hospitalization numeric rating scale (NRS) pain score, and recorded data to allow calculation of their FPSA scores. This sample differs slightly from our prior report due to inclusion of three additional patients who were excluded from the earlier report due to incomplete data.

### Patient safety, institutional review

Assessments were shared with treating physicians immediately, if they wished to use them to guide daily patient care. We submitted this study to the VCU IRB, and it was ruled as an exempt survey study for quality improvement in 2018.

### Procedures measures

Methods, including procedures, have been described previously.^7^^,^^8^ Briefly, procedures consisted of nursing staff providing paper surveys to patients for self-assessment, then collecting them for analysis and interpretation by the team. Patients were asked to complete procedures daily at approximately the same time of day. Patients were instructed to rate their pain and functional status for that specific day only, not based on the previous day’s experiences, to avoid recall bias and ensure day-specific assessment. Measures were collected on the day of admission, and daily if still admitted, up to day 17 (the maximum hospital LOS). The FSPA required an ordinal response of 1 to 5 to 10 items. (score range 10 to 50).^7^^,^^8^ The assessment was evaluated and found to be on a health literacy grade of one and was translated into Spanish, Arabic, and Chinese. We used the 11-point (0-10) numeric rating scale (NRS), which can be administered verbally, by phone, or from a distance. NRS pain intensity ratings are ordinal, not interval like the Visual Analog Scale.^10^ That is, the intensity distance between any two ratings is not the same.^11^ Length of stay was calculated, using hospital insurance claim records, as day of discharge minus day of admission. ED stays were excluded.

### Analysis

All hospitalization records for the sample were arranged so that each day’s pain score and FSPA scores could be concatenated in a daily sequence starting on the day of admission through the maximum hospital LOS, 17 days. We performed Principal Component Analysis (PCA) and built Partial Least Squares (PLS) models to explore the relationship between LOS, pain intensity, and the FSPA. Additionally, we built a Generalized Linear Model (GLM) to quantify the impact of changes in pain intensity on LOS. The statistical significance level was set at *P* < .05. The means of NRS pain intensity scores, same-day FSPA scores, and average LOS were calculated. The change in NRS pain intensity was obtained by calculating the difference between NRS on the day of admission versus the day of discharge. Since the ten items of the FSPA were highly correlated,^7^^,^^8^ principal component analysis (PCA) for factor selection and partial least square (PLS) model were used to analyze the relationships between LOS, individual FSPA items, and total FSPA score, and likewise the relationship between NRS, FSPA items, and total FSPA score. Correlation between LOS and change in NRS was analyzed using a generalized linear model (GLM), with LOS specified as the dependent variable. A Gamma distribution with a log link function was chosen due to the positively skewed and positive numeric sign of LOS, which is common for count-like or duration-based outcomes. Using the Gamma distribution avoids issues associated with using linear regression on heteroscedastic or non-normally distributed residuals. The change in NRS from admission to discharge was entered as the independent variable. Proc Pls in SAS didn’t give 95% confidence intervals (CIs) around the percent contribution of predictors to the variance in the outcome variable. We determined that Proc Reg in SAS did give CIs. However, we believed that Proc Reg was not a proper method for analysis of these data.

Further, we didn’t attempt power analyses to determine sample size prior to this study, since we didn’t have a pre-determined target effect size for trying to predict LOS or NRS pain intensity before data collection. We also did not conduct a post-hoc power analysis to determine whether the study was adequately powered to detect modest associations between FSPA scores, pain intensity, and LOS, since the results suggest that would be futile. All analyses were done with SAS 9.4. and JMP pro-16.

## RESULTS

**Figure 1.**
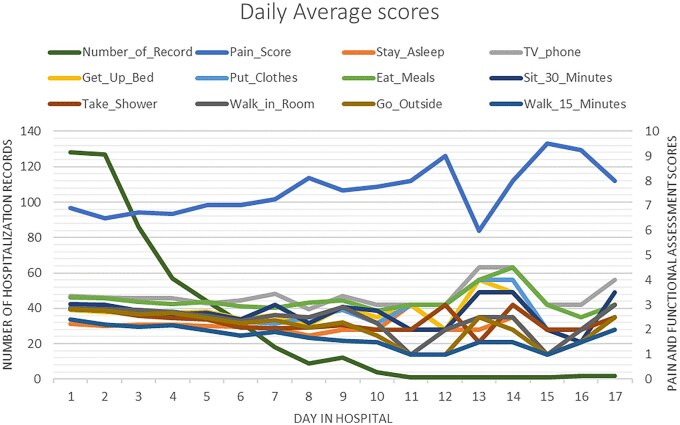
Number of patients assessed on each day post admission (left Y axis), and daily average of NRS and items of the FSPA (right Y axis). Abbreviations: FSPA, Functional Status-based Pain Assessment; NRS, pain intensity, numeric rating scale.

The population descriptors and the means of patient’s daily NRS and FSPA scores are shown in Table 1. The mean age was 34 ± 12.2 years, and 59.3% were female. A little over half of patients were genotype hemoglobin SS. The maximum LOS was 17 days. However, the number of FSPA and NRS observations diminished quickly after a LOS of 7 days. FSPA and NRS data were therefore censored at 7 days (Figure 1). From admission to discharge, NRS daily mean pain intensity actually increased, not decreased, though the increase was not statistically significant. FSPA scores actually decreased, rather than increased, from admission to discharge, indicating that pain-related physical function actually declined. This decrease was also not statistically significant.

**Table 1.** Participant demographic variables, average NRS, FSPA scores, and average LOS.

Characteristic	No.(%) (*N* = 91)
Sex	
Male	37 (40.7)
Female	54 (59.3
Age, mean ± Std	34 ± 12.21
Genotype	
SS	47 (51.6)
Sβ_0_	8 (8.8)
SC	20 (22.0)
Sβ^+^	6 (6.6)
SG Philadelphia	1 (1.1)
Unknown	9 (9.9)
Pain intensity, Mean (0-10 NRS)	
Admission day	6.89
Discharge day	6.28
FSPA total score, mean (0-50)	
Admission day	28.54
Discharge day	21.21
Length of stay, mean, days	3.36

Abbreviations: FSPA, Functional Status-based Pain Assessment; LOS, length of stay; NRS, pain intensity, numeric rating scale.

Principal component analysis of the relationships between LOS and baseline FSPA (day of admission) indicated that at baseline, only two factors had Eigen values > 1 (factor 1 eigen value = 5.59, factor 2 eigen value = 1.23), consistent with our previous study.^8^ The individual items of FSPA were each tested for their relationship to the variance in LOS (data not shown), but no factor was selected. Cross validation showed that all factors had root mean press >1, indicating the model predicted LOS worse than fitting the mean. Using all 10 items of the FSPA at baseline only explained 12.24% of the variance in LOS. Further, on the day of discharge, cross validation indicated only factor 1 had a root mean press <1 for FSPA. This one selected factor contributed 57.54% of the variance in FSPA, but only 5.69% of the variance in LOS. Thus, not only did FSPA at baseline not predict LOS, but also FSPA on the day of discharge did not predict LOS.

**Figure 2.**
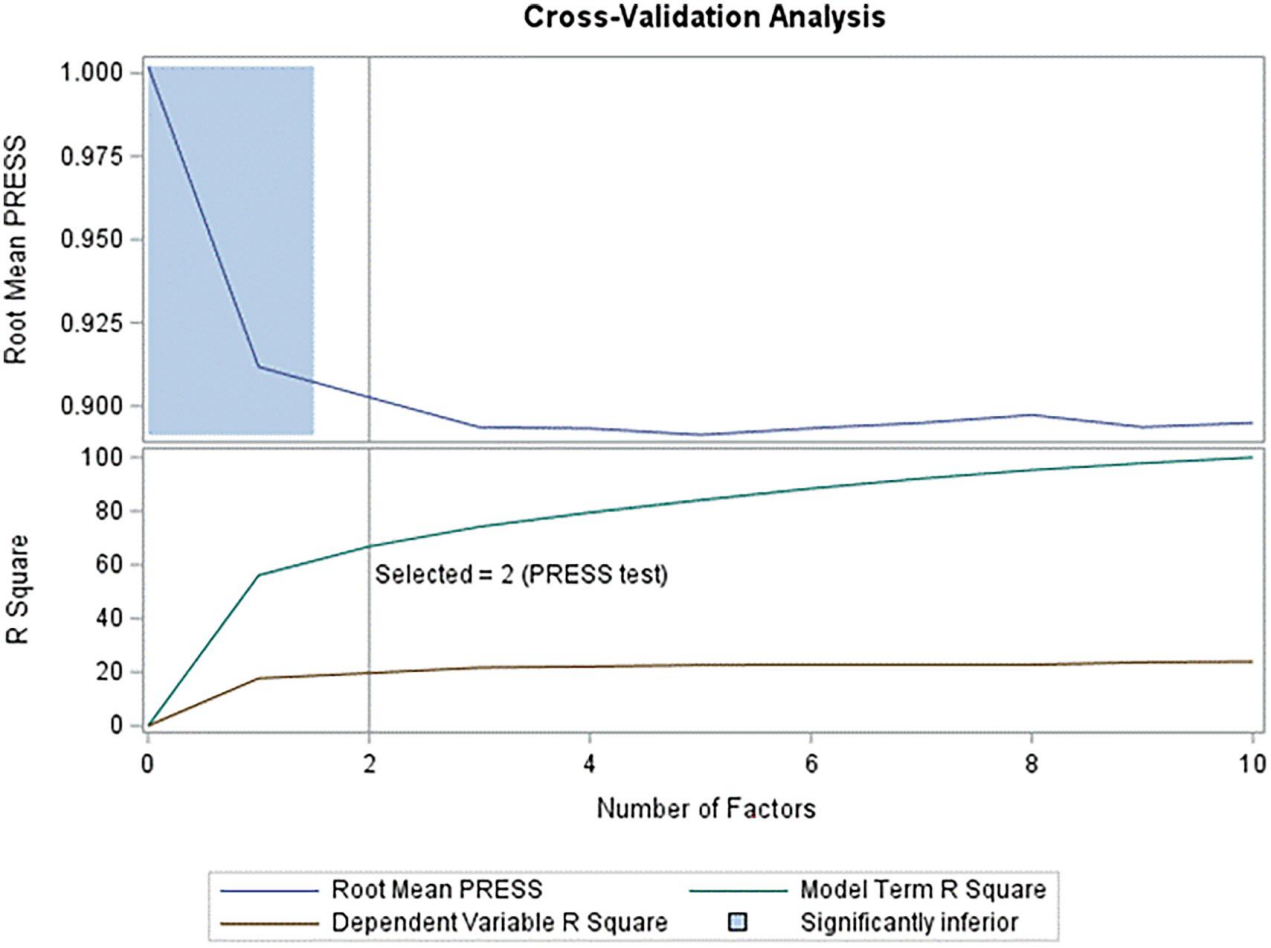
Cross-validation analysis of NRS and FSPA scores.

NRS pain intensity was analyzed as dependent variable with FSPA as independent variable. Two factors had root mean press <1 (Figure 2). These represented 66.81% of the variance in FSPA score, but only explained 19.66% of the variance in NRS (Table 2), indicating that FSPA score did not predict NRS well.

**Table 2.** Percent of variance in NRS accounted for by FSPA score, Principal Components analysis.

	**Model effects**		**Dependent variables**	
Length of stay (day of discharge)	Current	Total	Current	Total
Extracted Factor 1	57.53	57.53	5.69	5.69
**Pain sensitivity**				
Extracted Factor 1	56.06	56.06	17.68	17.68
Extracted Factor 2	10.74	66.81	1.98	19.66

Abbreviations: FSPA, Functional Status-based Pain Assessment.; NRS, pain intensity, numeric rating scale.

The generalized linear model type 3 analysis of LOS showed that the probability > Chi-Square for the regression *P*-value was 0.92 [no CI could be calculated], indicating that the change of NRS pain intensity from the day of admission to the day of discharge did not significantly influence LOS.

## DISCUSSION

We hypothesized we could demonstrate internal criterion validity of the FSPA for measuring daily function in hospitalized adults with SCD, by showing the FSPA was inversely related to unidimensional NRS over time, and by showing it partially predicted LOS in hospitalized adults with SCD. Further, we hypothesized that NRS pain intensity from admission to discharge would decline in hospitalized patients with SCD, and that FSPA scores would increase, offering two inversely related clinical indicators of readiness for discharge that patients and clinicians could rely on in daily discharge decisions for VOC.

Contrary to our hypotheses, FSPA scores did not relate (did not demonstrate criterion validity) to changes in daily diary pain intensity nor to changes in SCD LOS. We found the FSPA can not be used for one of the purposes we intended.

First, the two model factors which represented 66.81% of the variance in FSPA scores only explained 19.66% of the variance in NRS over the days sampled. Put another way, this model could only explain 19.66% of the variance in pain during hospitalizations, and so was essentially useless to bring up in conversations with patients as an accurate descriptor of their clinical state and potential readiness for discharge. Similarly, the FSPA score on the day of discharge explained only 5.69% of the change in LOS. Further, the model suggested that even discussion of pain intensity as a gauge of readiness for discharge with patients was useless.

Second, and perhaps most disappointing, the changes in both daily NRS and in daily FSPA within patients moved in the opposite directions that we hypothesized. Pain intensity increased, though not significantly, and FSPA declined, but not significantly. The two did not correlate. This helped explain the lack of statistical prediction of LOS by either of these two potential predictor variables.

Upon reflection, we offer the following explanations for our disappointing findings.

First, even if NRS and FSPA had varied as expected, it might have been extremely unlikely that the NRS or FSPA would show an association with LOS. Large practice variation and controversies in SCD pain management are well-known,^12^ and stem mainly from lack of evidence.^13^^,^^14^ In our practice, we have observed that LOS for a VOC depends on a plethora of variables. These variables enter the patient’s decision to seek care, the physician’s admission decision, and the daily discussion among SCD patients and their inpatient caregivers about readiness for hospital discharge.^6^^,^^15^ These variables include access to health insurance,^16^^,^^17^ quality of life,^18^ psychological adjustment,^19^^,^^20^ self-efficacy,^21–25^ depression and anxiety,^26^^,^^27^ stigma,^28^ alcohol abuse,^29^ sickle cell-specific stress,^6^^,^^30^^,^^31^ catastrophizing,^32^ somatizing,^33^ kinesiophobia (fear of movement),^34^ and maladaptive coping.^35–37^ These variables are usually left unmeasured during hospital stays for acute VOCs.

Second, we observe, and the literature supports, that often caregivers and patients negotiate, sometimes intensely and antagonistically, daily about admission and discharge decisions for a VOC.^26^^,^^29^^,^^32^^,^^33^^,^^38–45^ Along with trends in subjective NRS and functional status, trends in daily opioid use, as well as environmental, psychosocial, and economic drivers may each enter the negotiations. We speculate that clinicians and patients may interpret these variables differently, potentially leading to opposing opinions regarding readiness for discharge. Because we did not constrain patients or clinicians to use our measures, we should not be surprised by our failure to predict LOS using FSPA or NRS.

Still, we expected the NRS to be lower on discharge than on admission, since many often use it and rely on it as if it were determinant, and we expected the FSPA to be significantly higher (improved) on discharge than on admission, consistent with an improved patient at discharge. This finding would have meant that FSPA had a possible chance to correlate with daily NRS pain from admission to discharge.

Added to the above influences on admission and discharge decisions are institutional challenges such as bed availability and poor adult provider supply.^46^

Thus, a strong limitation of our study concerns our failure to measure and adjust for unmeasured confounding. For example, we did not ask patients to complete the Adult Sickle Cell Quality of life Measurement System (ASCQ-Me) survey, which was developed specifically for measuring some of these domains.^47^^,^^48^ We reasoned that the acute “state” influences on hospital stay and day-to-day pain would be independent of, and more influential than, “trait”, chronic biopsychosocial influences. Our results suggest we were wrong.

We expected that the FSPA would validate, because Zempsky’s Youth Acute Pain Functional Ability Questionnaire (YAPFAQ),^49^ the only inpatient functional pain assessment tool for SCD we found, validated in children when their function was measured sequentially post-operatively. YAPFAQ scores decreased over time showing good responsiveness to expected recovery.^50^ We conclude the YAPFAQ better predicts childhood inpatient SCD and post-operative behavior because childhood VOC behavior is less influenced by environmental, psychosocial, and economic drivers.

Can the FPSA scale be useful if co-measured with, or adjusted for, the confounding variables mentioned above? We are doubtful. We hypothesize that measurement and adjustment for these confounders would still not elevate the degree of influence of the FPSA and/or NRS during the in-hospital SCD pain experience in adults. However, given that the FPSA is correlated with same-day NRS, part of its future may rest in predicting mid-term health outcomes, for example in clinical studies of remittive agents meant to abort or acutely decrease VOCs. The ASCQ-Me and PROMIS measures have not been validated as sensitive to change in SCD for intervals shorter than 6 months.^47–53^ Perhaps the FSPA might be both multidimensional, that is, show a multi-factor structure, unlike the single-factor structure it demonstrates during a VOC, and sensitive to changes over intervals of weeks to months during a brief clinical trial or intervention. Future research should explore these hypotheses.

## Data Availability

The data presented in this study are available on request from the corresponding author. The data are not publicly available due to privacy.
